# Improving Discharge Voltage of Al-Air Batteries by Ga^3+^ Additives in NaCl-Based Electrolyte

**DOI:** 10.3390/nano12081336

**Published:** 2022-04-13

**Authors:** Yingying Gu, Yingjie Liu, Yunwei Tong, Zhenbo Qin, Zhong Wu, Wenbin Hu

**Affiliations:** 1 Tianjin Key Laboratory of Composite and Functional Materials, Key Laboratory of Advanced Ceramics and Machining Technology (Ministry of Education), School of Materials Science and Engineering, Tianjin University, Tianjin 300072, China; yingyinggu523@163.com (Y.G.); lyj_tju@163.com (Y.L.); tongyunwei@tju.edu.cn (Y.T.); qinzhb@tju.edu.cn (Z.Q.); wbhu@tju.edu.cn (W.H.); 2 Joint School of National University of Singapore and Tianjin University, International Campus of Tianjin University, Fuzhou 350207, China

**Keywords:** gallium, activation mechanism, Al-air battery, discharge voltage

## Abstract

The application of NaCl-based aluminum-air batteries is limited due to the passivation of the aluminum anode. In an effort to solve this problem, this work studied the influence of different concentrations of Ga^3+^ additives on the discharge behavior of Al in the NaCl electrolyte. The results of both experiments and theoretical calculations have shown that commercial purity aluminum could be significantly activated by Ga^3+^. Based on microstructure observations and electrochemical impedance spectroscopy, the influence activation mechanism of Ga^3+^ on the discharge behavior of commercial purity Al is clarified. The addition of Ga^3+^ biased the surface charge of aluminum along the activation direction, forming activation sites, and then destroyed the surface passivation film. Due to the formation of a gallium–aluminum amalgam, the Al-air battery had the best discharge characteristics in the electrolyte with 0.2 M Ga^3+^, and its discharge voltage reached 0.9734 V with a remarkable increase compared with that of NaCl solution (0.4228 V). Therefore, Ga^3+^ additive is a promising choice for NaCl-based Al-air batteries to improve their discharge voltage.

## 1. Introduction

Nowadays, the energy crisis and environmental pollution problems have become major focuses of global attention [1]. It is urgent to develop non-fossil fuel energy resources. Aluminum (Al) is the most abundant metal element in the earth’s crust and has a high electrochemical potential [2]. Thus, Al-air battery, as a new type of high specific energy chemical power source, has received special attention, owing to its high theoretical electrochemical capacity (2980 mAh g_Al_^−1^) and specific energy (8100 Wh kg_Al_^−1^), which are second only to the corresponding values of lithium-O_2_ battery [3]. Moreover, it does not produce toxic and harmful substances during operation, also being a recyclable product [4,5,6,7,8] which is considered a promising power source instead of fossil fuel.

Currently, Al-air batteries are mainly based on alkaline or neutral electrolyte. NaOH or KOH solution, as commonly used alkaline medium, could absorb CO_2_ from the air, leading to carbonation of the electrolyte, and then hindering air entry, causing mechanical damage and a decrease in battery performance [9]. Meanwhile, according to international regulations such as the Agreement Concerning the International Carriage of Dangerous Goods by Road (ADR), the Regulations Concerning the International Carriage of Dangerous Goods by Rail (RDI), and the International Maritime Dangerous Goods Code (IMDG Code), the transportation of batteries containing strong corrosive electrolytes is restricted due to the huge safety risks [10]. In comparison, a neutral electrolyte, such as NaCl solution and seawater, is safer to use with long-term stable operation, and this work is focused on Al-air batteries in a NaCl-based electrolyte. Unfortunately, the intrinsic protective oxide film on the surface of Al and its alloys would hinder the anodic dissolution, resulting in a discharge voltage as low as 0.3 V, unable to meet the practical demands [11,12,13,14]. Previous research was mainly related to anodic alloying to eliminate the passivation film by adding suitable alloying elements in the Al anode, such as Ga [15,16,17], In [15,18,19], and Sn [20,21]. The discharge voltage of Al–0.5Mg–0.1Sn–0.05Ga–0.05In (wt. %) anodes reached 1.389 V at 10 mA cm^−2^ in 2 M NaCl solution [19]. Ga generated active sites on the Al surface, decomposing the passivation film and effectively improving its electrochemical performance [16,22]. Even a small addition of Ga could also significantly increase the anodic current [15,23], and an Al–air battery assembled with Al–0.1 wt. % Ga anode showed competitive anodic efficiency (84.7%) with a relatively high discharge voltage of 0.86 V [15], indicating that Ga acted as an attractive activator for Al anode in Al-air batteries. Al anodic alloying has achieved a certain effect to improve battery performance. However, the process of Al alloying required high temperature melting of aluminum with the addition of elements for compounding, needing high energy consumption with a cumbersome preparation process. In addition, it still faced the outstanding problem of scale-up process when preparing large-scale samples [24]. In comparison, introducing Ga^3+^ additives directly into the electrolyte could simply and effectively improve the electrochemical activity of aluminum anode [25,26,27,28,29]. H.A. El Shayeb et al. [27] have found that the addition of Ga^3+^ activated the Al electrode with remarkable increases in the anodic current by an order of magnitude. According to the previous research and theoretical analysis, the addition of Ga^3+^ in the electrolyte has a positive effect, improving the discharge performance of Al-air batteries and especially promoting the anodic dissolution process of Al in the NaCl solution, but there is still a lack of corresponding research. 

In this work, different concentrations of Ga^3+^ were added to the NaCl electrolyte to explore its effect on improving the discharge voltage of Al-air batteries. The energy of Al in different solutions was calculated by Gaussian. The activation mechanism was also investigated by electrochemical measurements, Al anode morphology and structure characterization. The results show that it is feasible to improve the output voltage by using Ga^3+^ additive in the NaCl electrolytes.

## 2. Experiments

### 2.1. Battery Design and Fabrication

**Figure 1:**
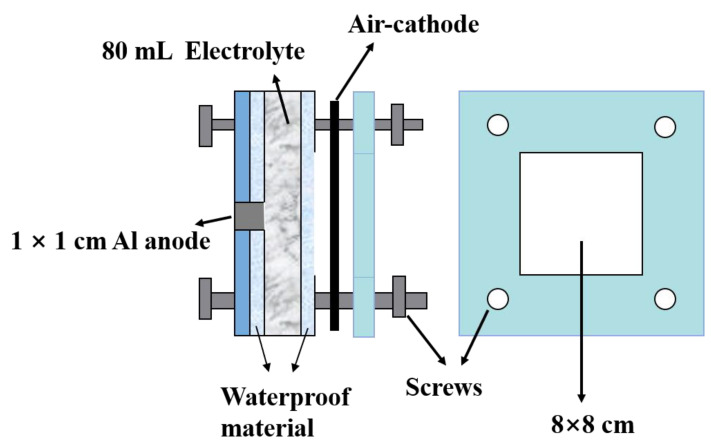
Schematic diagram of Al–air battery system.

A self-designed battery system is shown in Figure 1. The battery was subjected to the constant-current discharge tests (discharged at 10 mA cm^−2^ for 2 h) using a Neware battery test system. The distance between anode and air electrode is 1 cm. Because of its easy availability and low price, commercial purity aluminum (99.5 wt. %) was selected as the anode material with the active area of 1 × 1 cm with the composition as shown in Table 1. The test solutions (2 M NaCl solution free with the additive or with 0.05, 0.1, 0.15, 0.2, 0.3, or 0.5 M GaCl_3_ additive) were prepared using 99 wt. % anhydrous gallium chloride, sodium chloride (purchased from HEOWNS), and distilled water. The air cathode with the active area of 8 × 8 cm uses commercial MnO_2_ as the oxygen reduction reaction (ORR) catalyst.

The consumed anode weight was determined by weighting the anode before and after discharge, and the discharge capacity and energy density of the battery were calculated using the following formulas:

(1) $$\mathrm{Discharge}\;\mathrm{capacity}=\frac{It}{\Delta m}\;$$

(2) $$\mathrm{Energy}\;\mathrm{density}=\frac{UIt}{\Delta m}\;$$

where *I* is the discharge current (mA), *t* is the discharge time (h), Δ*m* is the weight loss (g), and *U* is the average output voltage (V).

The experiments were carried out at room temperature (25 ± 2 °C). All the above experiments were repeated at least three times to ensure reliable results.

### 2.2. Morphology and Structure Characterization

Scanning electron microscopy (SEM, JSM-7800F, JEOL, Tokyo, Japan) was combined with X-ray energy dispersive spectroscopy (EDS, OCTANE PLUS, EDAX, OR, USA) to capture morphological details with elemental analysis. Al anodes were discharged at 10 mA cm^−2^ in 2 M NaCl solution with different additives for 2 h, and samples were then rinsed with distilled water and dried in oven for 3 h to observe the surface topography. Then, the samples were sealed in epoxy resin to expose the cross-sectional structure. After gold spraying treatment, the cross-sectional morphology was observed, and elemental analysis was performed.

### 2.3. Electrochemical Tests

Electrochemical measurements were tested by a CHI660E electrochemical workstation using a classic three-electrode system. The reference electrode was a saturated calomel electrode (SCE), and the counter electrode was a platinum plate. Samples were mounted in epoxy resin exposed with 1 cm^2^ surface area as working electrode. Then, the working surfaces were ground with 400 to 1200 grit SiC sandpaper. All measurements were conducted at room temperature (25 ± 2 °C). The electrochemical impedance spectra (EIS) were measured at open circuit potential (OCP) after immersion for 1 h. The sweep frequency ranged from 100 kHz to 0.01 Hz with a sinusoidal voltage excitation of 10 mV amplitude. The EIS results were fitted using ZSimpWin software. Afterwards, a linear potentiodynamic sweep was performed in the range of ±5 mV OCP at a scan rate of 1 mV/s to determine the polarization resistance (*R_p_*). Subsequently, potentiodynamic polarization curves were measured at a scan rate of 1 mV/s to obtain the anodic and cathodic Tafel slopes (*b_a_* and *b_c_*), respectively. Then corrosion current density (*i_corr_*) was calculated by the Stern–Geary relation [30]:

(3) $$i_{corr}=\frac{b_{a}b_{c}}{2.303\times R_{P}\left(b_{c}-b_{a}\right)}\;$$

All the electrochemical measurements have been repeated at least three times to ensure the reliability of the testing results.

### 2.4. Simulation of Al in Different Solutions

Density Functional Theory (DFT) calculations were performed to investigate the instabilities of Al in in 2 M NaCl solutions without and with different contents of GaCl_3_ additives. In the case of this experiment, the ionic state of gallium atoms in the solution contains Ga^3+^ with a very small amount of unstable Ga^2+^, Ga(OH)^2+^, and GaO^+^ [31]. All calculations were performed by Gaussian 16 package.5 program, and the Solvation Model Based on Density (SMD) solvent model was used to describe the ionic solution. The energy of Al in NaCl aqueous solutions without and with the addition of 0.05, 0.1, 0.15, and 0.2 M GaCl_3_ were calculated, and the existence of GaCl_2_, Ga(OH)Cl_2_, and GaOCl was also considered.

Geometry optimization and frequency analysis were calculated at B3LYP functional [32] with 6-31G* basis sets. The SMD solvation modelling [33,34] was performed at m062X functional [35] with 6-31G* basis sets. Several parameters were considered to define the SMD model, including static dielectric constant of the solvent, dynamic dielectric constant of the solvent, surface tension at interface, and electronegative halogenicity.

## 3. Results and discussion

### 3.1. Battery Performance

**Figure 2:**
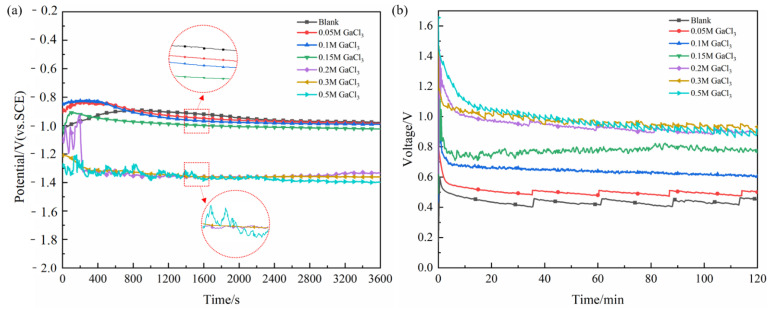
(**a**) Open-circuit potential of Al anode in 2 M NaCl electrolyte with different contents of GaCl_3_ additive. (**b**) Discharge curves of Al-air batteries in 2 M NaCl electrolyte at 10 mA cm^−2^ without and with different contents of GaCl_3_ additives.

Figure 2a shows the OCP curves of Al electrode, and it was found that the curves gradually shifted in the negative direction with the increase of Ga^3+^ concentration in the NaCl solution. The OCP value of 0.2 M Ga^3+^ additive decreased rapidly at around 200 s from the beginning of the experiment, forming an obvious descending step, subsequently reaching to much lower than without and with 0.05, 0.1, and 0.15 M Ga^3+^ additives ones. The OCP values of Al in NaCl electrolytes with 0.3 M and 0.5 M Ga^3+^ additives are not much different from that with 0.2 M Ga^3+^ additive, both around −1.35 V (vs. SCE). It is concluded that the addition of Ga^3+^ has an activation effect on the Al anode, especially for the electrolyte with greater or equal to 0.2 M Ga^3+^. In addition, it should be noted that the OCP curves show a fluctuating phenomenon as the concentration of added Ga^3+^ increases, which is attributed to the full activation of Al anode by high concentration additives and resulting in hydrogen evolution reaction (HER) between Al and water [19,36]:

(4) $$\mathrm{Al}+6H_{2}O\to 2\mathrm{Al}{\left(\mathrm{OH}\right)}_{3}+H_{2}$$

Figure 2b shows the typical discharge curves of the batteries at 10 mA cm^−2^, and the detailed values are listed in Table 2. In order to avoid the influence of unstable discharge at the beginning of the tests, the voltages in this paper are the average values of the discharge from 10 min to 120 min. A sample with a higher operating voltage usually has a stronger electrochemical activity and a better property. It can be found that the discharge voltage in NaCl was only 0.4228 V while the value increased with the addition amount of Ga^3+^ up to 1.0021 V, showing the similar trend with the results of OCP. Moreover, there are fluctuations in each curve, owing to the dynamic process of passivation film formation and dissolution on the surface of Al anode [37]. Besides the increase of discharge voltage, faster fluctuation frequency could also be found in the curves when Ga^3+^ raised more than 0.1 M, indicating the accelerating formation and rupture of the passivation film and resulting in the better activation performance. Although the discharge voltages with the addition of 0.3 M and 0.5 M Ga^3+^ additives are slightly higher than that with the addition of 0.2 M Ga^3+^, the pH value of the electrolyte is further reduced due to the hydrolysis of Ga^3+^, and the hydrogen evolution on the Al surface is very serious, resulting in their low discharge capacity and energy density. In general, the Al-air battery in the NaCl-based electrolyte with 0.2 M Ga^3+^ shows the relatively excellent discharge performance with the output voltage of 0.9734 V and the energy density of 1762.22 Wh kg_Al_^−1^. Excessive Ga^3+^ additive does not improve or even negatively affect the discharge performance of the battery, and it further increases the cost. Therefore, this manuscript has not studied the status with the addition of more than 0.2 M of Ga^3+^ additives in detail.

### 3.2. Discharge Morphology Analysis

**Figure 3:**
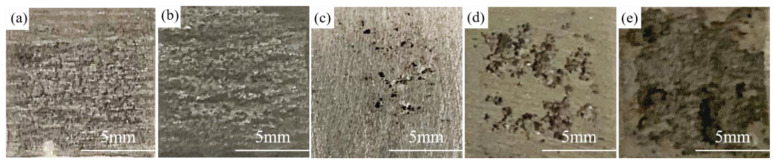
Optical images of Al anode being discharged at 10 mA cm^−2^ for 2 h in 2 M NaCl electrolyte (**a**) without and with different contents of GaCl_3_ additive: (**b**) 0.05 M, (**c**) 0.1 M, (**d**) 0.15 M, (**e**) 0.2 M.

**Figure 4:**
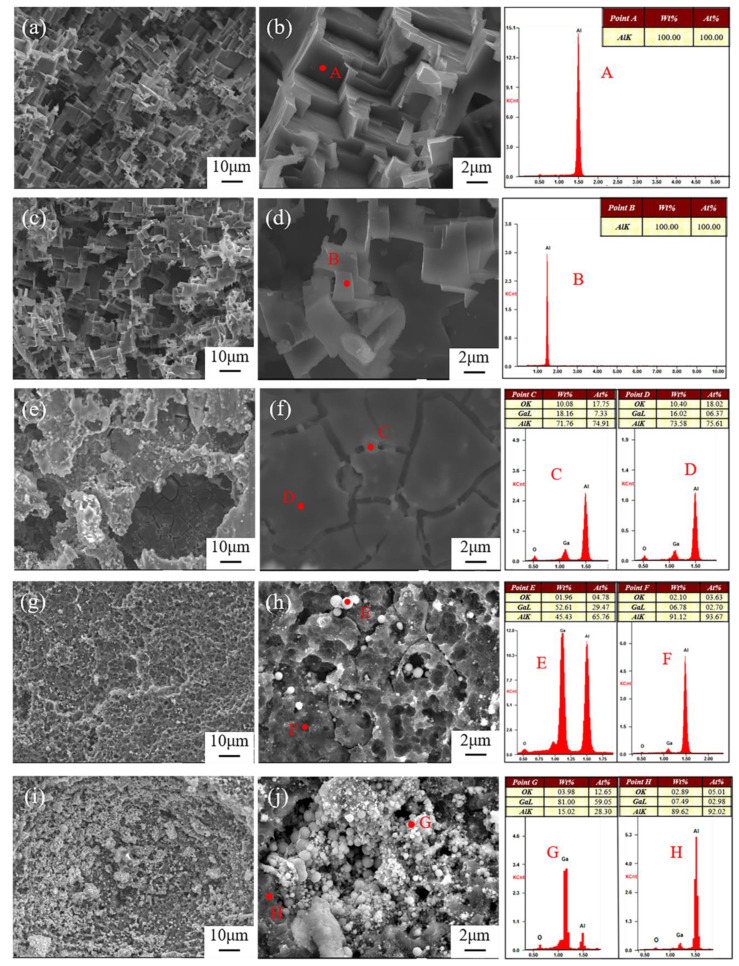
SEM images of Al anodes and the corresponding element contents of marked points after discharge at 10 mA cm^−2^ for 2 h in 2 M NaCl electrolyte (**a**,**b**) without and with different contents of GaCl_3_ additives: (**c**,**d**) 0.05 M; (**e**,**f**) 0.1 M; (**g**,**h**) 0.15 M; (**i,j**) 0.2 M.

The macroscopic morphologies of Al anode after being discharged at 10 mA cm^−2^ for 2 h are shown in Figure 3. There are many randomly distributed pits on the anode surface in the electrolyte free of additive (Figure 3a) and with 0.05 M Ga^3+^ (Figure 3b). When the addition of Ga^3+^ increased to 0.1 M, deeper corrosion pits could be seen in the local region, and its area continued to increase with the content of Ga^3+^, implying that the addition of Ga^3+^ was beneficial to the dissolution of Al anode. The detailed discharge morphologies of Al anode were further observed by SEM as shown in Figure 4. There is no obvious difference in the morphology of Al anode in the additive-free electrolyte and the electrolyte with 0.05 M Ga^3+^. The size of the pores as well as their distribution are relatively uniform. In the electrolyte with 0.1 M Ga^3+^, the morphology changed significantly with the shape of “cracked mud”. When the content of additive further increased to 0.15 and 0.2 M, spherical particles were found around the corrosion pits, and the surface film was decomposed by means of the activation effect of Ga^3+^.

According to the standard electrode potential φ^θ^(Ga^3+^/Ga) of −0.549 V, and φ^θ^(Al^3+^/Al) of −1.662 V [38], φ^θ^(Al^3+^/Al) < φ^θ^(Ga^3+^/Ga), and then, the following reaction occurs when the electrolyte contains Ga^3+^ where Ga^3+^ is reduced to metallic gallium:

(5) $$\mathrm{Al}+{\mathrm{Ga}}^{3+}\to {\mathrm{Al}}^{3+}+\mathrm{Ga}$$

**Figure 5:**
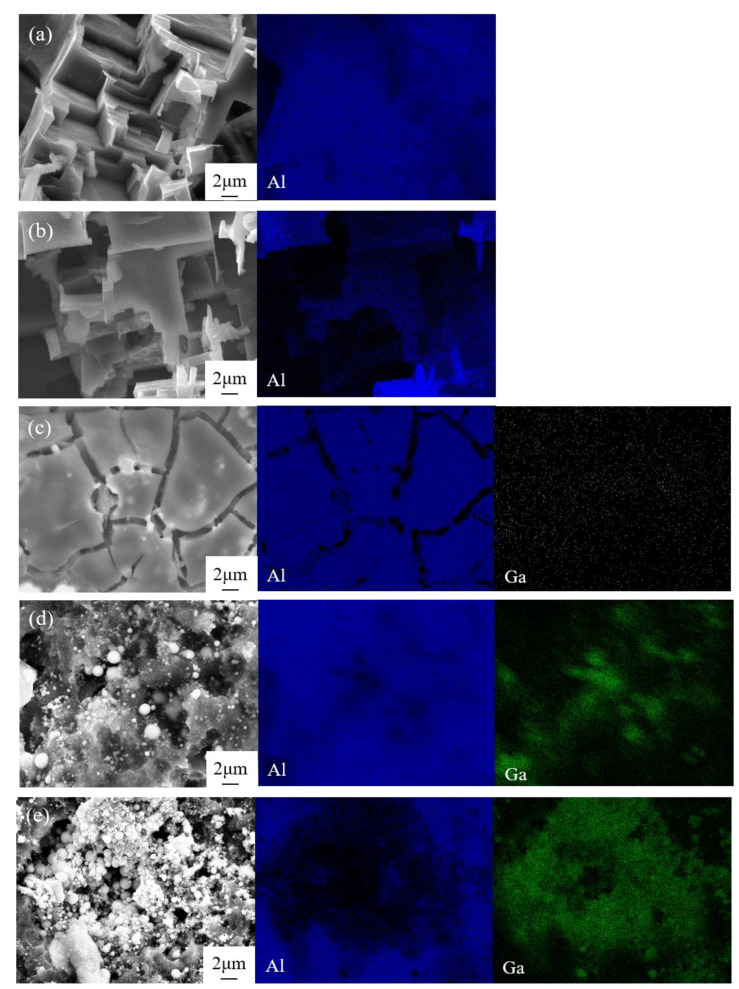
The elemental distribution of Al anode after discharge at 10 mA cm^−2^ for 2 h in 2 M NaCl electrolyte (**a**) without and with different contents of GaCl_3_: (**b**) 0.05 M, (**c**) 0.1 M, (**d**) 0.15 M, and (**e**) 0.2 M.

The above reaction process was clarified by the element analysis, and the corresponding results are shown in Figure 5. Elemental gallium was detected on the surface of Al anode after discharge except in the electrolyte free of the additive and also with 0.05 M Ga^3+^, indicating that Ga element was not generated according to reaction Formula (5) even though the reaction is thermodynamically feasible. It was attributed to the relatively slow kinetic process, and the electrochemical reduction of Ga^3+^ to metallic Ga with Al should occur with high concentrations of Ga^3+^ [25,29]. Previous work has shown that in 0.5 M NaCl solution containing 0.01 M Ga^3+^, more active behavior with metallic Ga was detected only after 9 days under natural immersion [29]. The induction period to generate metallic Ga has been quantitatively studied [39], and 0.56 wt. % GaCl_3_ solution has a nearly infinite induction period while 2.65 wt. % has an induction period of only 313 min. Owing to the deep undercooling characteristics of gallium, its nucleation process required a large degree of undercooling and sufficient time [40]. In the electrolyte with 0.1 M Ga^3+^, element Ga was detected on the Al anode surface as shown in Figure 4 and Figure 5c, and the content of element Ga at the crack (Point C) was slightly higher than that of smooth region (Point D), indicating that destruction of surface passivation film was promoted by high concentrations of element Ga. In addition, the pH value of the electrolyte decreased from 6.8 in 2 M NaCl to 1.2 with 0.2 M GaCl_3_, due to the hydrolysis of Ga^3+^ to generate hydrogen resulting in the decrease of discharge capacity as shown in Table 2.

(6) $$2H^{+}+2e^{-}\to H_{2}$$

**Figure 6:**
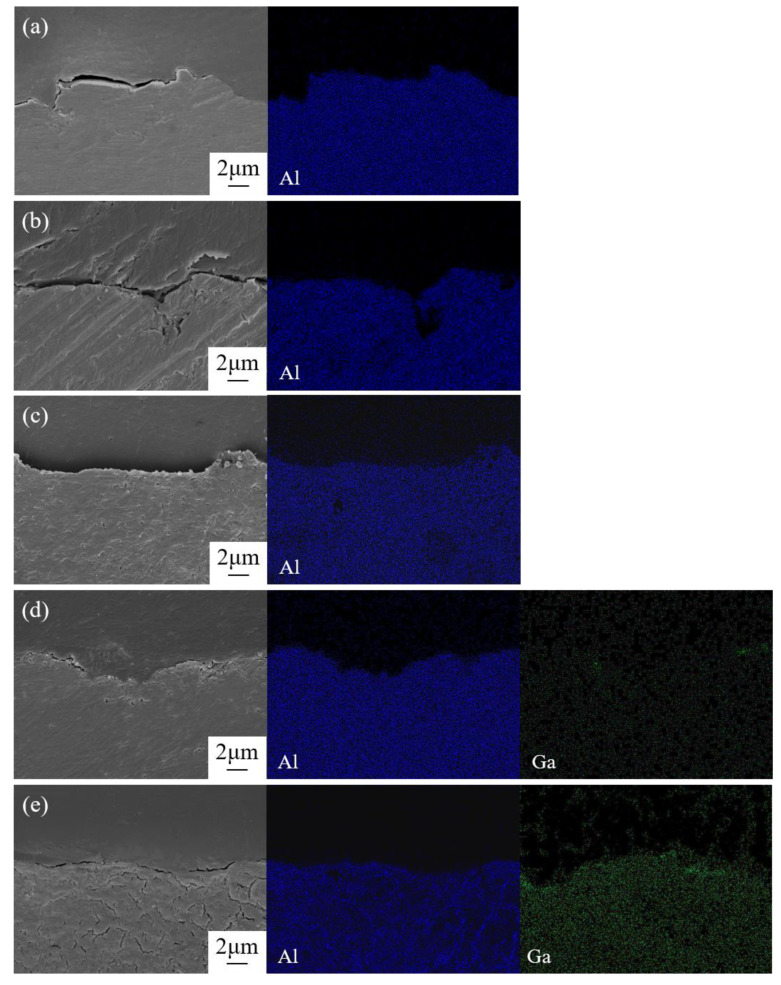
Cross-sectional images and the corresponding elemental distribution of Al anode after discharge at 10 mA cm^−2^ for 2 h in 2 M NaCl electrolyte (**a**) without and with different contents of GaCl_3_: (**b**) 0.05 M, (**c**) 0.1 M, (**d**) 0.15 M, and (**e**) 2 M.

The cross-sectional morphology and the corresponding element distribution of Al anode after discharge in different electrolytes are shown in Figure 6. It was found that only the sample in the electrolyte with 0.2 M Ga^3+^ exhibited a significant difference with others, where longitudinal cracks extended into the matrix owing to the grain boundary infiltration phenomena [41]. It is well known that the melting point of metallic Ga is only 29.8 °C, and the exothermic reaction (5) could easily lead to the transformation of metallic gallium from a solid state to a liquid one. When the liquid gallium contacted with solid Al, the liquid metal could infiltrate and diffuse along the Al grain boundaries to form an Al-Ga alloy by means of inter-atomic diffusion [41]. Previous studies have confirmed that the alloying elements only presented in solid solution form exhibited the activating effect [42]. Both Ga and Al belong to the third main group, and they are easy to form solid solutions owing to their similarity in structure. Because a large amount of metallic gallium was generated in the electrolyte with 0.2 M Ga^3^^+^, it was easier to form a solid solution during the infiltration process, and an amalgam reaction occurred to generate an aluminum–gallium amalgam to achieve the activation effect of Al anode.

(7) Al+Ga→Al(Ga)

### 3.3. Electrochemical Properties

**Figure 7:**
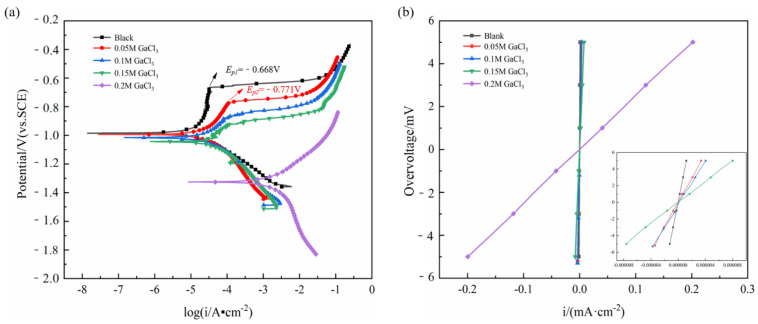
(**a**) Potentiodynamic polarization, (**b**) linear potentiodynamic sweep curves of Al in 2 M NaCl electrolyte without and with different content of additives.

Figure 7a shows the cyclic polarization curves of Al in the electrolyte with different content of additives, and the corresponding corrosion potential (*E_corr_*) and Tafel slopes (*b_a_*, *b_c_*) are listed in Table 3. The polarization resistance (*R_p_*) was determined from the slopes of linear polarization plots shown in Figure 7b. Then, corrosion current density (*i_corr_*) was calculated according to Equation (3), and the values are listed in Table 3. 

The anodic branches exhibit different polarization behaviors, and passivation regions can be clearly seen on the anodic branches of the electrolyte free of additive and with 0.05 M Ga^3+^. At the end of the passive region, the sudden increase in current density is caused by pitting corrosion, and the corresponding potential is called the pitting potential (*E_p_*) [19]. Compared with the value in the electrolyte without additive, the passivation zone was significantly shortened with a more negative *E_p_* by the addition of 0.05 M Ga^3+^, attributed to the breakdown of the surface passivation layer which was related to the deposition process of Ga^3+^. It can be seen from Figure 7b that *R_p_* decreased with the increasing concentration of Ga^3+^, implying the gradual diminished corrosion resistance [43], and the component with 0.2 M Ga^3+^ additive exhibited the lowest value of *R_p_* and *E_corr_* with the highest *i_corr_*, indicating the best activation ability came from the deposition of metallic Ga at specific active sites of Al and the formation of aluminum-gallium amalgam, which inhibited the generation of passivation film on the Al surface to improve its activity [26,44].

**Figure 8:**
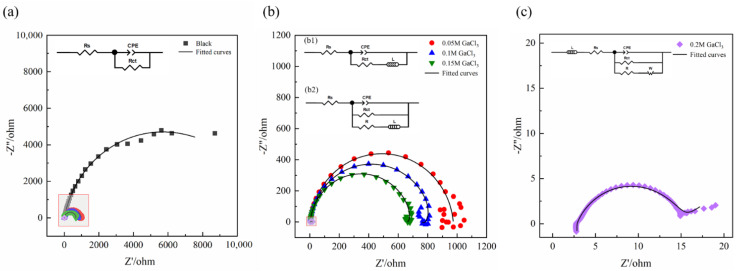
EIS of Al in 2 M NaCl electrolyte (**a**) without and with different contents of GaCl_3_ additives: (**b**) 0.05, 0.1, and 0.15 M; (**c**) 0.2 M.

In order to further understand the activation effect of Ga^3+^ additives on Al, EIS measurements were also carried out, as shown in Figure 8. When in NaCl solution, the EIS curve has only one capacitive reactance semicircle as shown in Figure 8a. In the electrolyte with 0.05, 0.1, and 0.15 M Ga^3+^ additives, all the EIS curves have a capacitive reactance semicircle and an indistinct inductive reactance semicircle. When the addition of Ga^3+^ increased to 0.2 M, there was a high frequency inductive semicircle, a capacitive semicircle, and a diffusion capacitive semicircle. In general, the diameter of the semicircle decreases with the increasing concentration of Ga^3+^, suggesting the gradual enhanced activity of Al.

The equivalent circuits are shown in the inset images of Figure 8. Among them, Figure 8(b1) is the equivalent circuit of aluminum in 0.05 M GaCl_3_, while Figure 8(b2) is the one in 0.1 M and 0.15 M GaCl_3_. *R_s_* is the solution resistance, *R_ct_* is the charge transfer resistance, *CPE* represents the non-ideal capacitance combination of double-charge layer and oxide film capacitance, and *W* represents the Warburg element. In the electrolyte with 0.05 M Ga^3+^ additive, there is no metallic gallium on the surface of Al, and thus the activation of Al is mainly dependent on Cl^-^. Ga^3+^ can bias the surface charge of Al along the activation direction, attract a large amount of Cl^-^ enrichment, generate more activation sites, and promote the dissolution of the passivation film [29]. The inductance *L* in Figure 8(b1) represents the relaxation behavior of pitting active sites and adsorbed intermediate corrosion products [45]. When the addition of Ga^3+^ increased to 0.1 or 0.15 M, deposition of Ga occurred; *R* represents the resistance due to the deposition of metal gallium, and the inductive reactance *L* in Figure 8(b2) is derived from the adsorption behavior of metallic gallium. The fitted electrochemical parameters are listed in Table 4, and the value of *R* in 0.15 M GaCl_3_ is greater than that of 0.1 M GaCl_3_, indicating the increasing deposition amount of metal gallium. With the further increase of Ga^3+^, the value of *R* significantly decreases owing to the generated aluminum–gallium amalgam. In addition, the value of charge transfer resistance (*R_ct_*) gradually decreases with the increase of Ga^3+^ additive. Especially for the 0.2 M Ga^3+^, its value is only about 1/20 of that with the addition of 0.15 M Ga^3+^, again proving that the formation of aluminum–gallium amalgam significantly improved the activity of Al. In the NaCl solution with 0.2 M Ga^3+^ additive, the inductance *L* represents the inductance due to the hydrogen evolution behavior on the Al surface, illustrating the occurrence of reaction (4) [46]. At low frequencies, the EIS curve was at an angle of about 45° to the axis, and the Warburg impedance indicated that the reaction process is controlled by diffusion owing to the faster Al anodic dissolution process induced by the Ga^3+^ activation [15]. 

### 3.4. Energy Analysis

The energy of Al in different solutions was calculated by Gaussian, and the results were shown in Table 5. The calculation energy represents the energy of 1 mol Al in different solutions. It can be found that the addition of Ga^3+^ increases the energy of Al, which could be further raised with the increase of Ga^3+^ concentration. It means that Ga^3+^ in solution can improve the electrochemical activity of Al, and with the increase of Ga^3+^ concentration, the electrochemical activity of Al also increases, which theoretically supports our previous research.

### 3.5. Activation Mechanism by Ga^3+^ Additives

**Figure 9:**
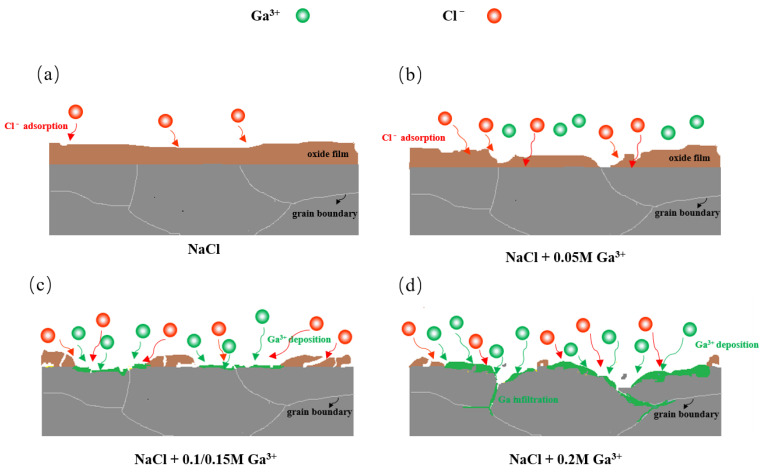
Activation mechanism of Al in the 2 M NaCl electrolyte (**a**) without and with different contents of GaCl_3_ additives: (**b**) 0.05 M, (**c**) 0.1 and 0.15 M, (**d**) 0.2 M.

The activation mechanism of Ga^3+^ additives on Al is proposed in Figure 9. When Al is discharged in the NaCl solution, the reaction is described as follows [47]:

$$\mathrm{Anode}:\mathrm{Al}-3e^{-}\to {\mathrm{Al}}^{3+},$$

(8) $$\mathrm{Cathode}:O_{2}+4H^{+}+4e^{-}\to 2H_{2}O$$

Since Al is easily oxidized, an oxide film forms on the surface, and the following reactions occur [48]:

(9) $$\mathrm{Al}+2H_{2}O\to \mathrm{AlOOH}+3H^{+}+3e^{-}$$

in which the AlOOH is Al_2_O_3_·H_2_O. When Cl^−^ exists, it competes with water molecules for adsorption on the surface of the oxide film. The attack of Cl^−^ leads to the dissolution of Al, while the reaction with water promotes the surface passivation. Adsorbed Cl^−^ reacts with Al in the oxide lattice [49]:

$${\mathrm{Al}}^{3+}\left({\mathrm{in}\;\mathrm{Al}}_{2}O_{3}\;\cdot \;H_{2}O\right)+{\mathrm{Cl}}^{-}\to \mathrm{Al}{\left(\mathrm{OH}\right)}_{2}\mathrm{Cl}$$

(10) $${\mathrm{Al}}^{3+}\left({\mathrm{in}\;\mathrm{Al}}_{2}O_{3}\;\cdot \;H_{2}O\right)+2{\mathrm{Cl}}^{-}\to \mathrm{Al}{\left(\mathrm{OH}\right)}_{2}{\mathrm{Cl}}_{2}$$

When adding 0.05 M Ga^3+^ additive, Ga^3+^ biased the surface charge of Al to the activation direction, attracted Cl^-^ to adsorb on the surface of Al [29], increased the number of sites for reaction (10) to occur, and promoted the dissolution of the surface oxide film. Therefore, Al anode could be activated even without the formation of metallic Ga when adding a small amount of Ga^3+^ with 0.05 M.

When Ga^3+^ concentration was greater than or equal to 0.1 M, it underwent redox reaction (5) with Al, decomposing the oxide film with cracks and then generating metallic Ga, and activating Al as shown in Figure 9c. When 0.2 M Ga^3+^ was added, however, a large amount of metallic Ga was produced in the corrosion pit. The low melting point metallic gallium induced an amalgam reaction, similar with that of metal Hg withAl to generate an aluminum–gallium amalgam [26], which detached the oxide film and avoided repassivation [26,44], resulting in the full activation of Al anode.

At the same time, in addition to the fact that the activated aluminum is prone to hydrogen evolution reaction [19,36], due to the liquid gallium infiltrates into the aluminum lattice along the grain boundary, the aluminum is embrittled and peeled off [44], which also led to the low discharge specific capacity (shown in Table 2) of the Al-air battery in the electrolyte with 0.2 M GaCl_3_. The improvement on the specific capacity of Al-air batteries will be the focus of our follow-up work by multicomponent additives.

## 4. Conclusions

1. The addition of Ga^3+^ additive in the NaCl electrolyte significantly activated the Al anode, which increased the Al-air battery discharge voltage from 0.4228 V to 0.9734 V with 0.2 M GaCl_3_.
2. In NaCl solution with 0.05 M Ga^3+^ additive, the addition of Ga^3+^ biased the surface charge of Al towards the activation direction, induced the enrichment of Cl^-^ and then generated many active sites, thereby promoting the dissolution of the passivation film.
3. When the added Ga^3+^ concentration was greater than or equal to 0.1 M, Ga^3+^ reacted with Al at specific activation sites, decomposing the oxide film and then generating metallic gallium to activate the Al anode.
4. In the NaCl solution with 0.2 M Ga^3+^ additive, the generated metallic gallium formed an aluminum–gallium amalgam with Al, which detached the oxide film and avoided repassivation, resulting in the full activation of Al anode.

## Figures and Tables

**Table 1:** Chemical composition of commercial purity Al.

Alloy	Al	Fe	Si	Ti	Ca	K	V	Cl	S
Conc.(%)	99.513	0.157	0.134	0.087	0.046	0.023	0.019	0.013	0.007

**Table 2:** Discharge performance of Al-air batteries in 2 M NaCl electrolyte at 10 mA cm^−2^ without and with different contents of GaCl_3_ additives.

Additives	Voltage(V)	Discharge Capacity (mAh g_Al_^−1^)	Energy Density (W·h·kg_Al_^−1^)
Blank	0.4228	2530.79	1028.44
0.05 M GaCl_3_	0.4939	2458.03	1063.77
0.1 M GaCl_3_	0.6329	2242.90	1428.10
0.15 M GaCl_3_	0.7869	1993.28	1685.18
0.2 M GaCl_3_	0.9734	1582.42	1762.22
0.3 M GaCl_3_	1.0019	1370.32	1635.20
0.5 M GaCl_3_	1.0021	1162.79	1428.57

**Table 3:** Corrosion parameters of Al in 2 M NaCl electrolyte without and with different content of additives.

Additives	*E_corr_* (V vs. SCE)	*R_p_* (Ω cm^2^)	*b_a_* (mV dec^−1^)	*b_c_* (mV dec^−1^)	*i_corr_* (μA cm^−2^)
Blank	−0.984	4133.5	338.2	−143.0	10.6
0.05 M GaCl_3_	−0.995	1474.4	244.4	−160.3	28.5
0.1 M GaCl_3_	−1.016	1332.1	209.3	−171.8	30.8
0.15 M GaCl_3_	−1.042	658.2	178.0	−166.7	56.8
0.2 M GaCl_3_	−1.324	25.0	134.5	−305.5	1622

**Table 4:** Impedance fitting values of Al in 2 M NaCl electrolyte without and with different content of GaCl_3_ additives.

Additives	*R_s_* (Ω cm^2^)	*CPE* (Ω^−1^ cm^−2^ s^n^)	*n*	*R_ct_* (Ω cm^2^)	*R* (Ω cm^2^)	*L* (H cm^2^)	*W* (Ω^−1^ cm^−2^)
Blank	7.613	3.813 × 10^−5^	0.88	11410	—	—	—
0.05 M GaCl_3_	3.338	7.217 × 10^−5^	0.94	970.1	—	9.178 × 10^−2^	—
0.1 M GaCl_3_	2.878	7.804 × 10^−5^	0.93	833.5	8848	9.402 × 10^−3^	—
0.15 M GaCl_3_	2.700	1.081 × 10^−4^	0.94	690.8	16130	2.430 × 10^−4^	—
0.2 M GaCl_3_	2.826	2.263 × 10^−3^	0.74	37.60	18.64	1.269 × 10^−6^	0.2910

**Table 5:** Energy and energy changes of 1 mol Al in 2 M NaCl solution without and with different additives.

Additives	*E* (kJ)	Δ*E* (kJ)
Blank	−636,344.57	0
0.05 M GaCl_3_	−636,341.16	3.41
0.1 M GaCl_3_	−636,338.79	5.78
0.15 M GaCl_3_	−636,335.91	8.66
0.2 M GaCl_3_	−636,331.97	12.60

## Data Availability

The data presented in this study are available upon request from the corresponding author.
